# A Case of Acute Intermittent Porphyria Leading to Severe Disability in a Young 21-Year-Old Female

**DOI:** 10.7759/cureus.34757

**Published:** 2023-02-08

**Authors:** Adil Ramzan, Jenny Jia Ling Cao, John S Frazer, Penelope Stein, Shakeel Ahmad

**Affiliations:** 1 Stroke Medicine, Whipps Cross Hospital, London, GBR; 2 Endocrinology and Diabetes, Peterborough City Hospital, Peterborough, GBR; 3 Cardiac Care Unit, Peterborough City Hospital, Peterborough, GBR; 4 Intensive Care Unit, Peterborough City Hospital, Peterborough, GBR; 5 Hematology, King's College Hospital NHS Foundation Trust, London, GBR; 6 Acute/Internal Medicine, Peterborough City Hospital, Peterborough, GBR

**Keywords:** porphyria and disability, aip delay in diagnoses and disability, aip and disability, aip, acute intermittent porphyria

## Abstract

Acute intermittent porphyria (AIP) is a rare metabolic disorder that is challenging to diagnose and treat. Symptoms are nonspecific and severe acute attacks may be life-threatening. This is a case of a previously healthy 21-year-old woman diagnosed with an acute attack of AIP following recurrent hospitalizations with undiagnosed abdominal pain over a 12-month period with gradual onset of motor neuropathy which resulted in complete paralysis and respiratory failure. Through our case, we will highlight the challenges in AIP diagnosis and management, its potential severity, and how an early diagnosis could have prevented severe disability.

## Introduction

Heme binds to four globin chains to form hemoglobin, an important component of red blood cells. Heme is derived from porphyrins and several enzymes play a key role in the synthesis of heme [1]. If any of the enzymes is defective the precursors start to accumulate and result in an acute attack [2]. The type of porphyria depends on the type of affected enzyme. There are several types of porphyria, including acute intermittent porphyria (AIP), hereditary coproporphyria, variegate porphyria, and aminolevulinic acid dehydratase deficiency (ALAD) porphyria. In addition to these types, porphyria cutanea tarda is the most common type of cutaneous porphyria. Cutaneous porphyrias, variegate porphyria, and ALAD porphyria present with blistering and scarring of the skin in addition to the other symptoms. Porphyria symptoms are nonspecific and severe acute attacks may be life-threatening. AIP is the most common type of all porphyrias and is caused by an autosomal dominant variant in the gene coding for hydroxymethylbilane synthase (HMBS), also known as porphobilinogen synthase (PBG synthase), a key enzyme in heme synthesis. The consequent build-up of toxic heme precursors, especially alpha-aminolevulinic acid (ALA) and PBG, clinically manifests in a wide spectrum of neurovisceral signs and symptoms due to their toxic effects on nerve fibers. Delays in recognizing and treating attacks may lead to serious complications including predominantly motor axonal neuropathy, seizures, psychosis, or hyponatremia [2]. Due to the rarity and non-specific presentation of this condition, diagnoses can be challenging. This case illustrates a young lady who was previously well and had no co-morbidities and presented to hospitals recurrently with abdominal pain. She was seen in two different hospitals which added to the time to diagnosis. This case also demonstrates how severe AIP can be if treatment is delayed. With the help of this case, it is highlighted how important it is to keep this condition in mind in young patients especially females, with unexplainable severe abdominal pain as there is a risk of severe disability.

## Case presentation

**Figure 1:**
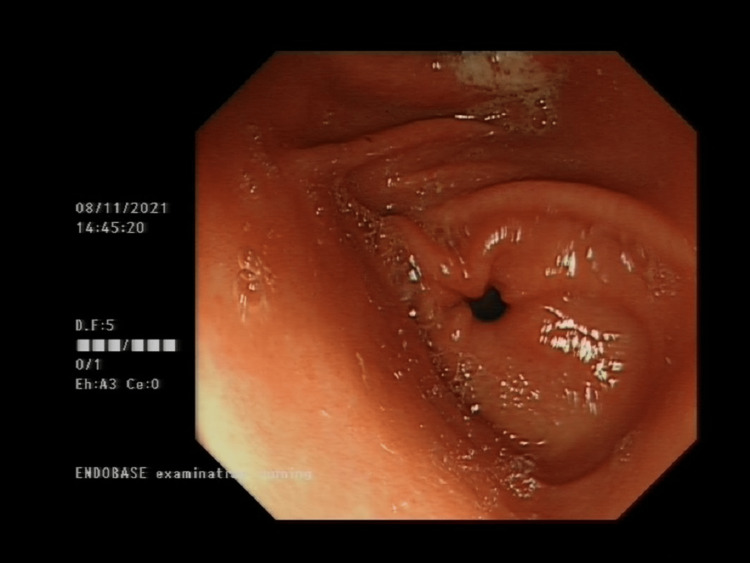
Oesophagogastroduodenoscopy The figure shows gastritis. There is no evidence of peptic ulcer disease as a cause of her recurrent abdominal pain.

A 21-year-old woman presented with a one-week history of severe, constant abdominal pain with dysuria. She was evaluated at the emergency department of her local hospital eight times over the previous 12 months with abdominal pain. In addition, she was also admitted to a different hospital where she had specialized scans, including computed tomography (CT) abdomen and pelvis, which were normal. Previous blood tests, including complete blood count, C-reactive protein, liver and renal function tests, and electrolytes, were all unremarkable. Physical examination and routine blood tests including C-reactive protein, full blood count, electrolytes, liver, and renal function tests were consistently unremarkable. She also underwent esophagogastroduodenoscopy (Figure 1), which showed gastritis. She was discharged with analgesics, a proton pump inhibitor, and sometimes antibiotics including nitrofurantoin for urinary tract infections (UTIs). Her social history was significant for smoking and cannabis use. She had been referred to the mental health team for a possible eating disorder, having had reduced oral intake for the past six months, weight loss of 10kg in two months with a body mass index (BMI) of only 14.5.

Two months before the hospital admission, which led to the diagnosis, she developed upper and lower limb weakness with recurrent falls leading to bruising of upper and lower limbs. Her family history was negative, as well as her past medical history. She was tachycardic on initial observations and hypertensive during her hospital stay. Her abdomen was soft and non-tender. She had weakness in the right upper arm, consistent with motor neuropathy, she had decreased strength against resistance in the right arm. There was no sensory deficit. Glasgow Coma Scale (GCS) score was 15/15. On admission, routine blood tests showed hyponatremia with sodium of 121 mmol/L but were otherwise unremarkable with normal inflammatory markers, renal function, cortisol, and thyroid-stimulating hormone (TSH). Urine culture was negative. Sodium fell to 114 after commencing intravenous (IV) 0.9% NaCl. Her plasma and urine osmolalities were 249 and 660 mOsmol/kg, respectively. Her urinary sodium was 165 mmol/L. Plasma osmolality, urine osmolality, and urinary electrolytes suggested the syndrome of inappropriate secretion of antidiuretic hormone (SIADH).

**Figure 2:**
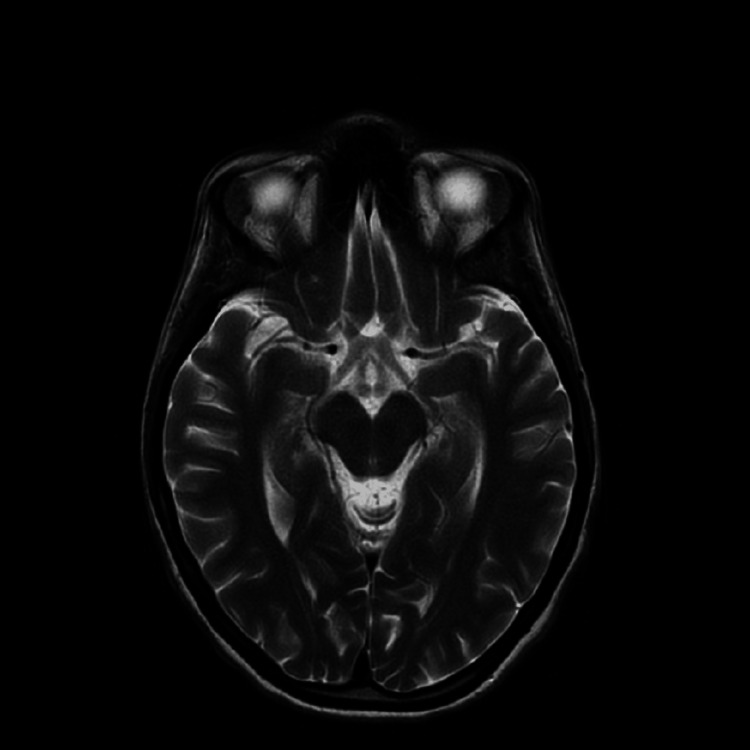
MRI brain MRI brain of the patient is negative for any evidence of infarction, hemorrhage, or tumor as a cause of her motor weakness. MRI: magnetic resonance imaging

**Figure 3:**
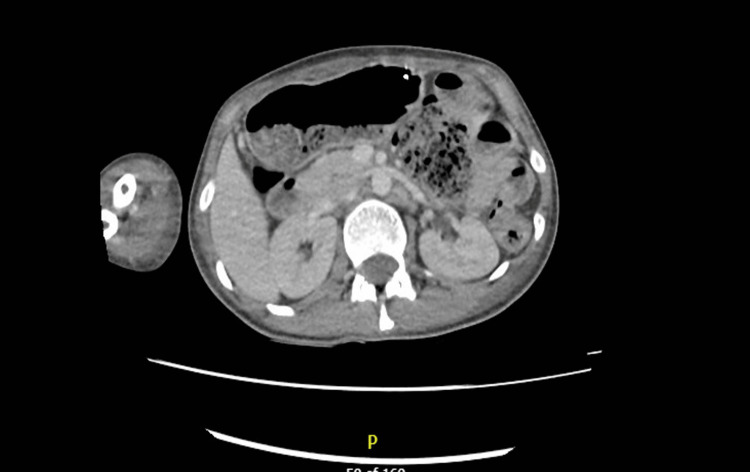
CT abdomen and pelvis with contrast CT abdomen and pelvis were negative for any evidence of acute pancreatitis, ascites, or inflammatory bowel disease as a cause of her abdominal pain. CT: computed tomography

**Figure 4:**
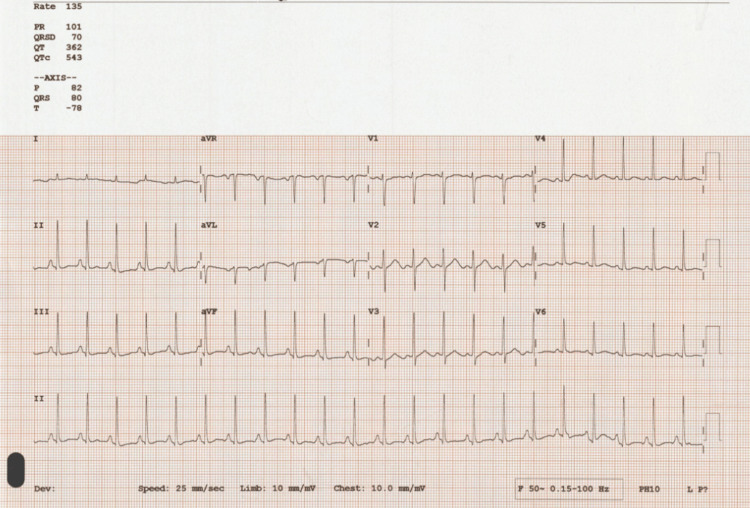
ECG ECG demonstrates sinus tachycardia with 135 beats per minute. The corrected QT interval (QTc) for tachycardia is 543 ms which is prolonged (normal range 360-460 ms). ECG: electrocardiogram

At this point, considering a young female with a history of recurrent abdominal pain associated with hyponatremia, neuropathy, and neuropsychiatric symptoms a possible diagnosis of AIP was considered [2]. The urine PBG screen was positive, and the sample was sent to a reference center for quantification. Her magnetic resonance imaging (MRI) head (Figure 2) excluded intra-cranial pathology and a CT abdomen and pelvis with contrast was unremarkable as well (Figure 3). Due to tachycardia (Figure 4), prolonged corrected QT interval (QTc) with chest pain and hyponatremia, she was transferred to the cardiac care unit (CCU), and these changes resolved after the correction of electrolyte imbalance.

On the sixth day of admission, PBG quantification confirmed a very high level of 86 μmol/mmol creatinine (reference range 0-1.5 μmol/mmol creatinine), and she was subsequently managed in collaboration with a porphyria specialist. Unfortunately, she had already deteriorated with reduced GCS of 7/15, type 1 respiratory failure [3], upper and lower limb weakness, and acute kidney injury. She was moved to the intensive therapy unit (ITU) where she was intubated and put on a ventilator under sedation. She also required vasopressor support. Haem arginate treatment was started to suppress her porphyrin levels. An initial weaning attempt in the ITU revealed that she had persistent bilateral motor neuropathy, more on the right side, with respiratory compromise. She was not able to move her legs and arms as well as had lost her head control. She was re-intubated, and later on, a tracheostomy was done. In addition to her peripheral motor neuropathy, she also developed swallowing difficulties which were later confirmed with the help of videofluoroscopy. She was commenced on total parenteral nutrition.

She had recurrent acute attacks over the next four weeks, and these were managed with four-day courses of haem arginate. Her recurrent porphyria attacks were felt to be delaying her neurological recovery, and she started monthly treatment with subcutaneous givosiran at the recommendation of a porphyria specialist.

She continued to have supportive management, including intensive physiotherapy sessions, and after 37 days of ITU stay, she was finally stepped down to a respiratory ward with a tracheostomy in situ. At this point, the patient had regained her head control and shoulders control and was able to flex her hips against gravity. She could communicate verbally but remained bed-bound as she was unable to bear weight. She had no further porphyria attacks, and her abdominal pain had settled. After almost three months of acute inpatient care, the patient’s tracheostomy was reversed, and she was transferred for inpatient neurorehabilitation. Her proximal strength of both upper and lower limbs muscles was 3/5, however, it was 2/5 distally at the time of discharge from the acute hospital.

## Discussion

Our case describes how easy it is to miss the diagnosis of AIP because it is rare and symptoms and signs are nonspecific. In the absence of obvious pathology, patients may be believed or thought to be exaggerating or even fabricating their pain. Missing or delaying the diagnosis of acute porphyria may result in catastrophic consequences. Acute attacks may occur in any of the subtypes of porphyria, including AIP, variegate porphyria, coproporphyria, and ALAD porphyria among others. AIP is difficult to diagnose due to the combination of its low prevalence and clinical heterogeneity.

**Figure 5:**
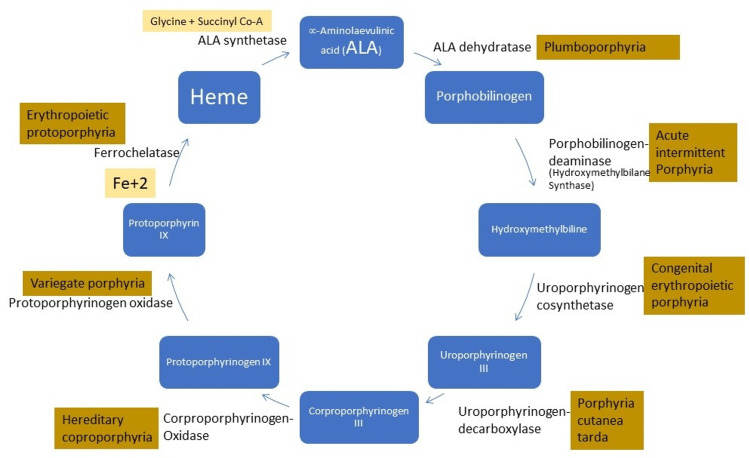
Heme synthesis pathway Image credit: Dr. Adil Ramzan

Figure 5 illustrates the heme synthesis pathway and different types of porphyria depending upon the affected enzyme. Alpha-ALA is derived from glycine and succinyl co-A in the presence of an enzyme called ALA synthetase which is then reduced to PBG by the action of another enzyme called ALA dehydratase. PBG is then converted to hydroxymethylbiline. PBG deaminase is required to catalyze this reaction and this enzyme is affected in AIP which results in the accumulation of the precursors including PBG and ALA which can be detected in urine and blood samples to help in making the diagnosis.

AIP is more common in females and typically presents in the late teens to early 30s. Symptomatic AIP has a prevalence of 5.4 per million in Europe [4]. The prevalence of pathogenic AIP gene variants is as high as one in 2000, but over 90% of people with a gene-variant will never have an acute attack which is due to a combination of genetic and environmental factors [5]. Penetrance is estimated at 20-50% in families with AIP but about 1% in the general population. Indeed, our patient did not have any family history of porphyria, but she had several possible attack triggers including caloric restriction, exposure to porphyrinogenic medications (she was given nitrofurantoin to treat UTI), smoking, recreational drug use, infection, and hormonal factors. Importantly our patient was under investigation for an eating disorder. She had lost weight, and her BMI was only 14.5 at presentation, which suggests that possibly she was avoiding eating because of her abdominal pains and that might have contributed to her severe attack. How avoiding eating can contribute to an acute attack is poorly understood [6]. When attacks do occur, the nonspecific presentation compounds the diagnostic challenge. However, the combination of abdominal pain (particularly when recurrent) with hyponatremia and neurological manifestations in a young adult is strongly suggestive of acute porphyria [7].

Abdominal pain occurs in nearly all attacks [8]. Pain is severe and poorly localized and may also be felt in the back, legs, and chest. Other common clinical features include nausea, vomiting, constipation, hypertension, tachycardia, psychiatric symptoms, seizures (which may be associated with hyponatremia), peripheral motor neuropathy (sensory neuropathy is less common), encephalopathy, hyponatremia, and dark orange or red colored urine. It is essential to identify any precipitating factors and try to remove them. These include drugs. Many commonly prescribed drugs are porphyrinogenic and may trigger or potentiate an attack. The safety of medications in porphyria can be checked at http://www.drugs-porphyria.org. Other triggers include hormonal factors in women (including the luteal phase of the menstrual cycle), alcohol, stress, reduction in calorie intake, and smoking. Triggering elements present in our patient included nitrofurantoin prescribed for a possible urine infection, severe calorie restriction, smoking, recreational drug use, and stress.

Routine investigations should include a complete blood count, C-reactive protein, renal and liver function tests, and urine and plasma osmolarities (if hyponatremia). Depending upon presentation, electroencephalography (EEG) and MRI head can be considered. The essential investigation to diagnose or exclude an acute attack of porphyria is a urine PBG concentration which requires a random light-protected sample. Further specific investigations, including urine, plasma, and fecal samples (all light protected) should be sent for porphyrin biochemistry to diagnose and differentiate between the four types of acute hepatic porphyrias [9]. Genetic studies provide confirmation.

An attack of AIP may be life-threatening if not treated promptly. In general management of an acute attack involves supportive and specific treatments. Mild attacks may resolve with supportive management, but careful observation is needed as attacks can progress from mild to severe very quickly. A high carbohydrate diet may be helpful if patients tolerate oral intake however IV dextrose in water solutions may precipitate or aggravate hyponatremia and should be avoided. Haem arginate remains the treatment of choice for all severe porphyria attacks, Haem arginate works by reducing the synthesis of δ-ALA, but in patients with recurrent episodes causing significant disability and requiring repeated courses of haem arginate, then givosiran may be indicated [10-12]. Givosiran is a new gene-silencing therapy, which works by reducing the levels of 5-ALA synthase, an enzyme required for the production of δ-ALA. In our patient, givosiran was highly effective at preventing further attacks that would have delayed neurological recovery [13,14]. Along with these, pain management, respiratory support, intensive physiotherapy, nutritional support, medication review for safety in porphyria, mental health support [15], and patient and caregiver education should be continued in parallel. Organizations support groups such as the National Acute Porphyria Service, National Organization for Rare Disorders, and the British Porphyria Association provide valuable resources [16].

Our case is a clear example of how severely AIP can affect a patient’s mental and physical status and emphasizes how important is to make early diagnoses that could effectively have prevented significant disability.

## Conclusions

In conclusion, AIP can lead to severe disability if diagnoses or treatment is delayed. The most common presentation is non-specific abdominal pain and initial investigations are usually negative. Young females of post-pubertal age are more commonly affected. Patients may present with weight loss due to a decrease in oral intake in an attempt to avoid abdominal pains. In suspected cases, light-protected samples of urine, blood, and feces should be sent for qualitative and quantitative analysis. If there is a suspicion of AIP, medications should be reviewed for their safety profile in porphyrias. Haem arginate is the first-line treatment and it should be commenced as soon as possible. Givosiran, a novel agent, can be considered in severe and refractory attacks after consultation with a porphyria specialist. Early diagnosis and commencement of treatment may prevent severe complications.
